# The Effects of Daytime Psilocybin Administration on Sleep: Implications for Antidepressant Action

**DOI:** 10.3389/fphar.2020.602590

**Published:** 2020-12-03

**Authors:** Daniela Dudysová, Karolina Janků, Michal Šmotek, Elizaveta Saifutdinova, Jana Kopřivová, Jitka Bušková, Bryce Anthony Mander, Martin Brunovský, Peter Zach, Jakub Korčák, Veronika Andrashko, Michaela Viktorinová, Filip Tylš, Anna Bravermanová, Tom Froese, Tomáš Páleníček, Jiří Horáček

**Affiliations:** ^1^ National Institute of Mental Health, Klecany, Czechia; ^2^ Third Faculty of Medicine, Charles University, Prague, Czechia; ^3^ Czech Technical University in Prague, Prague, Czechia; ^4^ Department of Psychiatry and Human Behavior, School of Medicine, Center for the Neurobiology of Learning and Memory, University of California, Irvine, Irvine, CA, United States; ^5^ First Faculty of Medicine, Charles University, Prague, Czechia; ^6^ Embodied Cognitive Science Unit, Okinawa Institute of Science and Technology Graduate University, Okinawa, Japan

**Keywords:** neuroplasticity, EEG power spectra, antidepressant, psilocybin, sleep, Rapid Eye Movement sleep, Rapid Eye Movement latency, slow-wave (delta-wave) sleep

## Abstract

Serotonergic agonist psilocybin is a psychedelic with antidepressant potential. Sleep may interact with psilocybin’s antidepressant properties like other antidepressant drugs via induction of neuroplasticity. The main aim of the study was to evaluate the effect of psilocybin on sleep architecture on the night after psilocybin administration. Regarding the potential antidepressant properties, we hypothesized that psilocybin, similar to other classical antidepressants, would reduce rapid eye movement (REM) sleep and prolong REM sleep latency. Moreover, we also hypothesized that psilocybin would promote slow-wave activity (SWA) expression in the first sleep cycle, a marker of sleep-related neuroplasticity. Twenty healthy volunteers (10 women, age 28–53) underwent two drug administration sessions, psilocybin or placebo, in a randomized, double-blinded design. Changes in sleep macrostructure, SWA during the first sleep cycle, whole night EEG spectral power across frequencies in non-rapid eye movement (NREM) and REM sleep, and changes in subjective sleep measures were analyzed. The results revealed prolonged REM sleep latency after psilocybin administration and a trend toward a decrease in overall REM sleep duration. No changes in NREM sleep were observed. Psilocybin did not affect EEG power spectra in NREM or REM sleep when examined across the whole night. However, psilocybin suppressed SWA in the first sleep cycle. No evidence was found for sleep-related neuroplasticity, however, a different dosage, timing, effect on homeostatic regulation of sleep, or other mechanisms related to antidepressant effects may play a role. Overall, this study suggests that potential antidepressant properties of psilocybin might be related to changes in sleep.

## Introduction

Psilocybin (O-phosphoryl-4hydroxy-N, N-dimethyltryptamine) and its active metabolite psilocin (4-hydroxy-N, N-dimethyltryptamine) are the main psychoactive components of psychedelic mushrooms. In the central nervous system, psilocin acts as an agonist of serotonergic 5-HT1A and 5-HT2A/C receptors, leading to altered states of consciousness in humans (Tyls et al., 2014). Psilocybin doses of 0.04–0.43 mg/kg cause alterations in perception, cognition, and emotions, while also eliciting long-term changes in well-being and mood in both healthy and psychiatric subjects (Dos Santos et al., 2016; Korčák et al., 2019; Barrett et al., 2020). These long-lasting positive changes lead to an exploration of the therapeutic potential of psychedelics as well as the mechanisms underlying this potential. Because of its general safety, intermediate duration of action, and therapeutic potential in several neuropsychiatric disorders, psilocybin is currently the most intensely studied psychedelic in clinical trials (Sewell et al., 2006; Grob et al., 2011; Stebelska, 2013; Dos Santos, 2014).

Overall, there has been an increasing preclinical (Catlow et al., 2013; Baumeister et al., 2014) and clinical (Carhart-Harris et al., 2016; Bogenschutz and Ross, 2018) evidence for the antidepressant potential of psilocybin. Although exact mechanisms are currently unknown, it is generally believed to be attributed to either a direct action on 5-HT receptors or the psychological effects of acute intoxication (Carhart-Harris and Goodwin, 2017). It has been shown that serotonergic psychedelics including psilocybin via 5-HT2A receptors promote neuroplasticity (Ly et al., 2018), a fundamental mechanism of neuronal adaptation that is disrupted in depression (Carhart-Harris and Goodwin, 2017) and restored by antidepressant treatments including selective serotonin reuptake inhibitors (SSRIs), tricyclic antidepressants (TCAs) or electroconvulsive therapy (ECT) (Hayley and Littlejohn, 2013).

Synaptic plasticity and sleep display a close relationship. Specifically, slow-wave activity (SWA), a marker of the intensity of non-rapid eye movement (NREM) slow-wave sleep (SWS), is hypothesized to actively maintain synaptic homeostasis (Esser et al., 2007) and support memory functions dependent on neuroplasticity (Buzsáki, 1998; Huber et al., 2004; Marshall et al., 2006). According to the synaptic homeostasis hypothesis, SWA reflects changes in synaptic strength and contributes to the normalization of cellular homeostasis (Tononi and Cirelli, 2014). However, SWA expression is altered in patients with depression (Kupfer et al., 1984; Benca et al., 1997; Plante et al., 2012a; Plante et al., 2012b). Moreover, it has been proposed that decreased neuroplasticity in depression, as well as the efficacy of multiple depression treatments, may directly depend on SWA expression (Goldschmied and Gehrman, 2019; Rantamäki and Kohtala, 2020). Sleep is thus a plausible proxy to evaluate synaptic plasticity and its induction by psilocybin and other psychotropics *in vivo*.

Other sleep changes have been associated with the use of antidepressant drugs. The most consistent effects of SSRIs, TCAs, serotonin and norepinephrine reuptake inhibitors (SNRIs), and monoamine oxidase inhibitors (MAOIs) have been reported on REM sleep in terms of reduced duration and increased latency, both after acute and chronic use (Wilson and Argyropoulos, 2005). However, the effect of sedating drugs like antidepressants with antihistaminergic action, sedating TCAs, mirtazapine, mianserin, or drugs with a strong antagonistic action at serotonergic 5-HT2 receptors, like trazodone and nefazodone is less pronounced showing little or no effect on REM sleep (Wilson and Argyropoulos, 2005; Wichniak et al., 2017) while decreasing sleep latency, improving sleep efficiency and increasing SWS. NREM sleep changes such as increased delta sleep ratio and time spent in SWS have been observed after longer antidepressant treatment by sertraline (Jindal et al., 2003; Zhang et al., 2013) but were not shown after a short use of SSRIs (Wilson and Argyropoulos, 2005; Rasch et al., 2009; Tesler et al., 2013). As most antidepressants alter and normalize REM sleep and SWS changes in patients with depression, these sleep changes may be directly associated with the observed antidepressant effects (Wichniak et al., 2013; Wichniak et al., 2017; Rantamäki and Kohtala, 2020).

Serotonergic psychedelics such as LSD, dimethyltryptamine (DMT), and mescaline show sleep alterations including an increase in wakefulness and inhibition of REM and NREM sleep (Colasanti and Khazan, 1975; Kay and Martin, 1978). Moreover, Ayahuasca, another serotonergic psychedelic containing both DMT and MAOIs, caused a decrease in REM sleep duration and a trend to increase REM latency while enhancing SWA in the first sleep cycle after acute daytime administration (Barbanoj et al., 2008). Analogously, ketamine, a drug with rapid antidepressant effects and psychological side effects is very similar to serotonergic psychedelics. A sub-anesthetic infusion of 0.5 mg/kg ketamine has been shown to enhance SWS duration and SWA, promote memory and neuroplasticity, and increase the amount of REM sleep in depressed patients (Duncan et al. 2013a; Duncan et al., 2017).

Despite the fact, that psilocybin is currently the most intensely investigated psychedelic, the absence of any preclinical or clinical sleep data after acute administration of psilocybin represents a major knowledge gap related to its potential antidepressant effect. Therefore, the primary aim of our study was to evaluate changes in sleep associated with psilocybin administration. More specifically, the study aimed to identify how psilocybin changed both macro- and microstructural sleep parameters to study both traditional sleep stage measures as well as more detailed quantitative aspects of sleep by conducting whole-night polysomnography with 19 channels electroencephalography (EEG).

We hypothesized that, analogously to serotonergic antidepressants (e.g., SSRIs) and serotonin agonists, psilocybin would 1) prolong REM onset latency and 2) decrease the proportion of REM sleep. In line with tenants of the synaptic homeostasis hypothesis and findings from ketamine and ayahuasca sleep studies, we also expected that 3) psilocybin will increase SWA during the first sleep cycle where most SWA occurs (Barbanoj et al., 2008; Duncan et al., 2013a; Tononi and Cirelli, 2014). To study quantitative parameters of sleep in more detail, spectral power was examined during the whole night across multiple frequency bands during REM and NREM sleep stages. Lastly, as in ayahuasca research (Barbanoj et al., 2008), no changes in the subjective perception of sleep and no changes in objective sleep latency, total sleep time, and sleep efficiency were expected. For exploratory purposes only, ex post analyses were additionally conducted to control for possible gender differences. No gender differences were expected to occur.

## Methods

### Participants

All participants provided written informed consent before they entered the study. The study was approved by the Ethical Committee of the National Institute of Mental Health (NIMH-CZ), by the State Institute for Drug Control and as a clinical trial registered under the EudraCT No. 2012-004579-37.

Twenty healthy volunteers (10 women) aged 28–53 (mean = 36 ± 8.1 years) were enrolled in the present sleep experiment as part of a larger ongoing study (for details: EudraCT No. 2012-004579-37). Participants were included if they were free of regular use of medication, had no major medical or psychiatric condition present, no family history of psychotic disorder, no present sleep disorder, and met other criteria as previously specified in (Bravermanova et al., 2018). To control for the effect of female hormonal changes on sleep and mood, our women volunteers were tested at times not overlapping with their menses. Pregnant women were excluded per protocol and we did not include any menopausal or postmenopausal women. All participants were asked to abstain from psychotropic drug use during the period between the introductory interview and the experiment, excessive alcohol use leading to drunkenness for 1 week before the experiment. Participants were also asked to remain free of coffee, tobacco, and food intake in the morning before the experiment. One participant was excluded due to excessive daytime sleepiness (recognized during the placebo EEG session) and another two due to technical issues with polysomnographic recordings. Data from a total of 17 subjects were included in the final analysis.

### Drug Administration

All volunteers participated in two sessions, one with an inactive placebo and one with psilocybin (active drug) in a double-blind, placebo-controlled crossover design (for details see Bravermanova et al., 2018). Psilocybin was administered in 1 and 5 mg capsules in the morning of the experimental nights (around 9 am). The dose was adjusted according to body weight (approximately 0.26 mg/kg). The dose increased 1 mg by every 5 kg of body weight and was thus within the range of 15–22 mg, mean = 18.35 mg, s.d. = 2.21. For example, a person weighting 76–80 kg received a 20 mg dose of psilocybin. Psilocybin induced robust psychedelic effects with the test period lasting approximately 6.5–8 h during which the psychedelic effects dissipated. Participants came into the sleep laboratory with no psychedelic effects present. Repeat sessions occurred at a minimum of 28 days apart per protocol (mean = 49 days apart) after which any potential long-term effects of psilocybin on sleep had presumably disappeared.

### Polysomnography Recordings

In line with standards of good sleep research practice, all participants except 2 volunteers underwent an adaptation night at least 2 days before the beginning of the experiment (median of 7.5 days). Whole-night polysomnography, approximately 12 h after the placebo/psilocybin ingestion, was performed using a gel cap with 19 electrodes according to the 10/20 standard system with electrooculography (EOG), electromyography (EMG, three submental electrodes), and electrocardiography (ECG). Data were recorded using the Brainscope polysomnography system (M&I spol. s.r.o., Czech Republic) with a band-pass filter of 0.1–200 Hz and continuously digitalized at a sampling rate of 1 kHz. Data were downsampled to 250 Hz before further processing. Sleep scoring was provided visually by two expert scorers blinded to the experimental condition according to the AASM international criteria (Berry et al., 2015). The interrater agreement reached 87%, Cohen’s kappa was 0.82. In those recordings where the agreement did not reach at least 80% level, a third blinded scorer acted as a tie-breaker for inconsistent epochs.

### Sleep Assessment

After each experimental night, subjective and objective sleep characteristics were evaluated. Subjectively perceived sleep latency, sleep duration, sleep quality were quantified using 4-point Likert scales (one for “very poor,” four for “perfect”). Objective sleep latency, sleep duration, sleep stage durations, and sleep efficiency were computed.

An EEG spectral analysis was conducted using MATLAB (The MathWorks, Inc, United States) with FIR filtering set at 0.5–40 Hz. All recordings were visually checked by four experienced raters who manually rejected all 5-s epochs with any of the following: electrode artifacts, muscle, or eye movement artifacts. As a result, all NREM and REM sleep epochs were free of eye movements and related artifacts which could bias spectral estimates. A Fast Fourier transform (FFT) was then implemented in 5-s epochs with a 50% overlap utilizing a standard hamming window. Power spectra density for each 5-s artifact-free epoch was derived following FFT calculation by the squaring of its absolute values. Power spectra density was then averaged across epochs for each channel and by each sleep stage. Resulting values were then log-transformed and averaged across the following defined frequency bands: delta (0.8–4.6 Hz), theta (4.6–8 Hz), alpha (8–12 Hz), sigma (12–15 Hz), beta1 (15–20 Hz), beta2 (20–35 Hz) and absolute and relative power spectral values were computed.

For the SWA analysis in the first sleep cycle, each recording was visually checked and manually limited to the end of the first cycle. Spectral analysis was then applied as described above. To limit the number of comparisons, all 19 original derivations were aggregated into respective average frontal, central, parietal, temporal, and occipital areas, e.g., O1 and O2 channels were averaged into an occipital region. To directly compare SWA in the first sleep cycle and SWA across the whole night, all 19 original derivations were aggregated into an average electrode.

### Statistical Analysis

Based on data distribution paired-samples t-tests or Wilcoxon signed-rank tests were used to assess differences in all studied sleep characteristics following daytime placebo and psilocybin administration. EEG power spectral results were corrected for multiple comparisons using the Sidak test (Šidák, 1967) for both SWA analysis of the first sleep cycle and the whole night sleep analysis (in all frequency bands across all 19 derivations separately for each sleep stage). Effect sizes for both paired-samples t-tests and Wilcoxon signed-rank tests were computed using product-moment correlation coefficients (r) according to (Rosenthal, 1991). For explorative purposes, a series of two-way repeated measures ANOVAs was conducted ex post to find any possible gender differences, with experimental condition (psilocybin/placebo) as within subjects factor and gender (female/male) as between subjects factor. Any significant interactions between experimental condition and gender were followed by *post hoc* tests (Bonferroni tests). All statistical analyses were done using IBM SPSS Statistics 23 (IBM Corporation, United States) MATLAB software, and Statistica 13 (TIBCO Software Inc., United States).

## Results

### Effects of Psilocybin on Whole Night Sleep Stage Architecture

Sleep latency, total sleep time, sleep efficiency, and the number of sleep cycles were not significantly different in placebo and psilocybin conditions (Table 1). A significant increase in REM latency was found for the night after psilocybin administration, z = −1.66, *p* = 0.048 (1-tailed, uncorrected). The effect size was small (r = −0.28). Sleep architecture in terms of duration or proportion (% of total sleep time spent in the sleep stage) of sleep stages did not differ significantly in the drug vs. placebo conditions (Table 1). However, statistical trends for decreased R, 1, and N3 duration and increased N2 proportion were observed after psilocybin administration (uncorrected).

**TABLE 1:** Sleep macrostructure after daytime administration psilocybin and placebo (uncorrected).

	Placebo	Psilocybin	*p*-Values
Sleep latency^a^ (min ± SEM)	14.37 ± 2.37	21.68 ± 4.63	0.149
REM latency^a,b^ (min ± SEM)	82.62 ± 5.05	115.05 ± 12.23	**0.048**
Total sleep time (min ± SEM)	400.28 ± 11.33	388.00 ± 12.98	0.315
Sleep efficiency (% ± SEM)	88.78 ± 2.14	88.75 ± 2.31	0.989
Sleep cycles (no. ± SEM)	4.06 ± 0.22	3.88 ± 0.22	0.565
N1 (% ± SEM)	3.39 ± 0.39	2.66 ± 0.26	0.101
N2 (% ± SEM)	42.62 ± 1.94	46.74 ± 2.41	0.051
N3 (% ± SEM)	22.53 ± 1.08	20.68 ± 1.66	0.175
R (% ± SEM)^b^	20.24 ± 1.52	18.48 ± 1.04	0.148
WASO (% ± SEM)	11.22 ± 2.14	11.24 ± 2.31	0.994
N1 (min ± SEM)	15.25 ± 1.72	11.63 ± 1.17	0.080
N2 (min ± SEM)	193.17 ± 10.27	205.13 ± 11.6	0.216
N3 (min ± SEM)	100.90 ± 4.18	90.37 ± 6.68	0.078
R (min ± SEM)^b^	90.90 ± 6.58	80.75 ± 4.83	0.095
WASO (min ± SEM)	50.28 ± 9.35	48.75 ± 9.78	0.878

a Denotes for non-normally distributed differences, non-parametric tests used.

b Denotes for 1-tailed tests.

N1, Stage 1 NREM sleep; N2, Stage 2 NREM sleep; N3, Stage 3 NREM sleep; R, REM sleep; WASO, wake after sleep onset; SEM, standard error of the mean. Values highlighted in bold are values significant at 0.05 level (uncorrected).

#### Effects of Psilocybin on SWA during the First Sleep Cycle—A Proxy of Neuroplasticity

**FIGURE 1:**
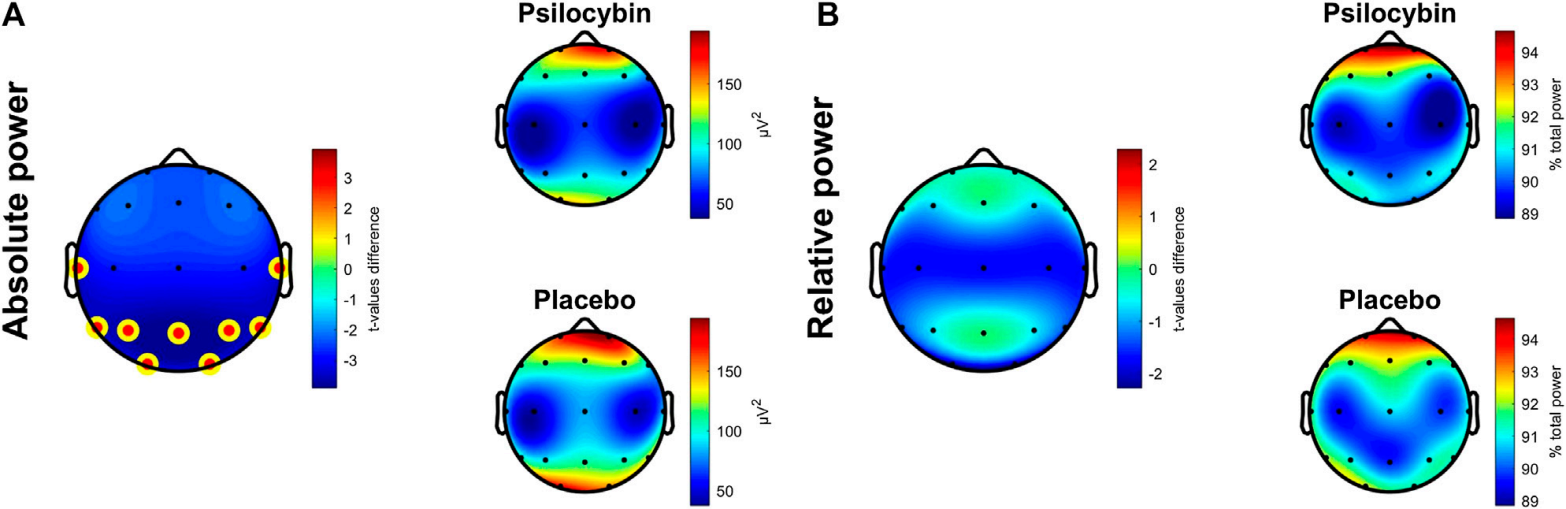
**(A)** Topographic plots of differences in t-values (Psilocybin–Placebo) in absolute delta power **(left)** and average absolute delta power in psilocybin **(left top)** and placebo **(left bottom)** condition during the first SWS cycle, significant over averaged parietal, temporal and occipital derivations (corrected). **(B)** Topographic plots as described in **(A)** for relative delta power with all differences non-significant. The yellow-red dot denotes areas significant at *p* < 0.01 (corrected).

Absolute delta power during SWS in the first sleep cycle was found to be significantly lower after psilocybin administration in comparison to placebo at the average electrode (t (16) = −3.57, *p* = 0.003, r = 0.67, medium effect), and locally at averaged frontal, central, parietal, temporal and occipital derivations. After correcting for multiple comparisons, a significant decrease remained at averaged parietal (t (16) = −3.93, *p* = 0.001), temporal (t (16) = −3.40, *p* = 0.004) and occipital (t (13) = −3.26, *p* = 0.006) derivations with large effect sizes (r = 0.70, 0.65, 0.67 respectively). In relative delta power a significant decrease was not observed at average electrode. However, it was locally observed only in averaged occipital derivations (t (13) = −2.29, *p* = 0.039, r = 0.54, large effect) in the psilocybin relative to the placebo condition (Figure 1), although a trend decrease in EEG relative delta power was also observed at the averaged central derivations in the psilocybin relative to the placebo condition (t (16) = −1.861, *p* = 0.081, r = 0.42, medium effect). After correcting for multiple comparisons, no local changes remained significant in relative spectral power.

#### Effects of Psilocybin on Sleep Microstructure—The Whole Night EEG Power Spectra

**FIGURE 2:**
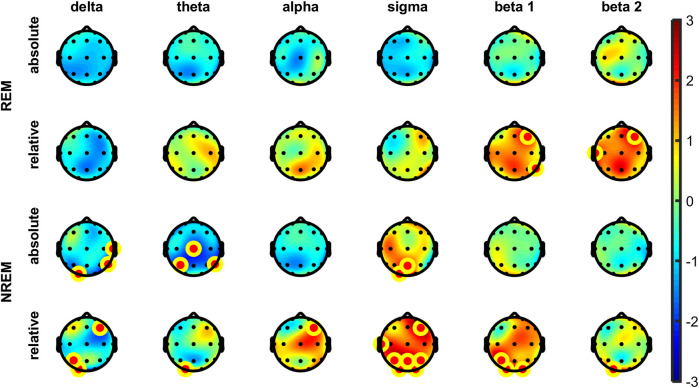
Topographic plots of differences in t-values (Psilocybin–Placebo) in absolute **(top)** and relative **(bottom)** spectral power for each frequency band in the REM **(top)** and NREM **(bottom)** sleep across the entire sleep period. The yellow-red dot denotes electrodes significant at *p* < 0.05 (uncorrected). No differences remained significant in both absolute and relative spectral power after correcting for multiple comparisons (Sidak method, all frequency bands, all 19 derivations).

The analysis of EEG power spectra revealed no significant differences in power spectral density during N1, N2, N3, or R sleep stages in either absolute or relative spectral power in any of the defined frequency bands. However, in NREM overall (N1-N3) some significant increases of relative but not absolute power were visible in sigma band frontally and parietally (i.e., F4, P3, P4). No other differences were observed in any other bands in NREM overall (Figure 2). After correcting for multiple comparisons, no changes remained significant in either absolute or relative spectral power in all frequency bands at all 19 derivations. For average electrode, a trend decrease after psilocybin administration in comparison to placebo was found in absolute delta power (t (16) = −1.87, *p* = 0.081, r = 0.42, medium effect) but not in relative delta power.

### Effects of Psilocybin on Subjective Sleep Measures

No significant changes in subjective total sleep time or sleep quality were observed (Table 2). However, significant results were found in subjective sleep latency where subjects perceived time to fall asleep as longer (10.5 min on average) during the night after psilocybin administration compared to placebo condition (t (18) = 2.39, *p* = 0.028). This effect was medium-sized, r = 0.49.

**TABLE 2:** Changes in subjective sleep parameters after psilocybin administration (uncorrected).

	Placebo	Psilocybin	*p*-values
Subjective sleep latency (min ± SEM)	18.75 ± 3.18	29.30 ± 4.87	**0.028**
Subjective total sleep time^a^ (min ± SEM)	430.00 ± 10.83	418.33 ± 13.60	0.408
Subjective sleep quality	3.29 ± 0.16	3.14 ± 0.21	0.480

Mean ± SEM (*n* = 18).

Values highlighted in bold are values significant at 0.05 level (uncorrected). ^a^Subjective sleep latency differences not normally distributed, non-parametric tests used.

### Gender Differences

Analyses revealed no main effects of gender nor significant interactions between gender and experimental condition in any of the sleep macrostructural properties (i.e., sleep latency, REM latency, total sleep time, sleep efficiency, sleep cycles, individual sleep stages in min and %). In SWA during the first sleep cycle, a significant main effect of gender was found in mean relative delta power (across all electrodes) suggesting that women had significantly higher relative delta power than men in our sample, irrespective of experimental condition, F (1, 15) = 4.584, *p* = 0.049. However, this effect was not found locally at frontal, central, parietal, temporal, and occipital electrodes (corrected). In subjective sleep parameters, only one significant interaction between gender and condition was found, F (1, 12) = 15.429, *p* = 0.002. Only men had significantly lower subjective sleep quality after psilocybin administration.

## Discussion

As expected, the main finding of this study is that psilocybin significantly increased REM sleep onset latency and showed a trend toward reduced REM sleep duration on the first night after administration. These results are in line with the effects of SSRI, SNRI, TCA, and MAOI antidepressants (Wichniak et al., 2017). Serotonergic agonists without antidepressant effect, e.g., fenfluramine (Myers et al., 1993) or pergolide (Tagaya et al., 2002) do not show such an effect. Shortened REM sleep onset latency, increased REM sleep duration and increased REM density have been previously considered as biological markers of depression (Palagini et al., 2013), and their normalization was associated with the reduction of depressive symptoms (Wichniak et al., 2017). Thus, induced changes of REM sleep onset latency observed in the present study may be related to the antidepressant effects of psilocybin where corresponding doses of psilocybin (i.e., 10–25 mg) were administered (Carhart-Harris et al., 2016; Carhart-Harris et al., 2017; Carhart-Harris and Goodwin, 2017).

Contrary to our expectations, psilocybin significantly decreased absolute delta power during SWS in the first sleep cycle on the night after psilocybin administration. From the neurochemical perspective, our finding corresponds with the research on fenfluramine and other 5-HT_2_ receptor agonists that decrease SWA (Myers et al., 1993; Monti, 2011). However, this result contradicts ketamine research, which suggests increased SWA at the first night after treatment (Barbanoj et al., 2008; Duncan and Zarate, 2013; Duncan et al., 2017). The difference can be attributed to different neurochemical effects of ketamine (Kapur and Seeman, 2002) or the shorter pharmacokinetics after intravenous infusion of ketamine in comparison to the oral presentation of psilocybin (Zanos et al., 2018; Korčák et al., 2019) and thus to differing effects on the timing of plasticity processes. Concurrently, Catlow et al. (2013) showed that the effects of psilocybin on neurogenesis (the formation of new neurons, also related to sleep) are dose- and time-related. It is thus possible that a different dose or administration timing could affect the expression of SWS, similarly as in SSRIs (e.g., sertraline) that increase SWS after 12 weeks of treatment (Jindal et al., 2003).

An alternative explanation for the present findings may be that the acute effects of psilocybin cause a decrease in homeostatic sleep pressure. Altered sleep pressure may in turn reflect suppressed neural synchrony and/or recruitment of wider cortical neural networks, processes that underlie plastic changes related to synaptic homeostasis (Vyazovskiy et al., 2011; Tononi and Cirelli, 2014). Lastly, present SWA changes may also be explained by mechanisms other than neuroplasticity. SWA suppression could normalize abnormally increased SWS power in depression (Schwartz et al., 2001; Plante et al., 2012a) and could relate to the improvement of depressive symptoms as was previously shown using disruptive acoustic stimuli during SWA (Landsness et al., 2011). Future research should investigate whether psilocybin leads to SWA suppression in clinically depressed populations and whether SWA changes relate to the improvement of depressive symptoms.

To investigate qEEG changes of sleep after psilocybin administration in general, a set of power spectral analyses was conducted. On the first night after administration, psilocybin did not affect EEG power spectra in NREM or REM sleep stages robustly. The only differences were found in increases of relative power in the sigma band in NREM sleep overall. Although these differences did not remain significant after correction for multiple comparisons and could be associated with the decrease of SWA (Andrillon et al., 2011) in the present study, future research on larger samples is warranted. Sigma activity has been associated with sleep spindles which are believed to play an important role in sleep maintenance protection as well as in memory consolidation, synaptic plasticity (Astori et al., 2013), and depression (Nishida et al., 2016; Sesso et al., 2017; Hamann et al., 2019).

Congruently with our expectations, psilocybin did not significantly affect objective and subjective sleep continuity or overall subjective sleep quality on the first night after administration. The only parameter affected, a prolonged subjective sleep latency, was comparable to that observed after ayahuasca (Barbanoj et al., 2008). The tendency to the increased proportion of N2 sleep is in line with reported acute effects of SNRI, TCA (Wilson and Argyropoulos, 2005; Wichniak et al., 2017), and fenfluramine (Myers et al., 1993). This change could be related to decreases in REM and N3 sleep (Roehrs and Roth, 2010).

Despite no gender differences in sleep macrostructure were found, the analysis of relative SWA delta power during the first sleep cycle identified higher SWA in women, a finding consistent with literature (Mourtazaev et al., 1995; Ehlers and Kupfer, 1997; Carrier et al., 2001). Interestingly, the gender differences interacting with psilocybin administration were found only in subjective sleep quality. Women generally report more subjective sleep problems than men (Reyner et al., 1995; Lindberg et al., 1997; Groeger et al., 2004) which is an opposite trend as shown after psilocybin administration. It is possible that psilocybin worsens subjective quality in men. However, this result must be treated with caution given low sample size.

Several limitations of the current study should be mentioned. First, results could be influenced by the lack of statistical power given our small sample size. However, our sample size is comparable to other pharmaco-sleep research studies (e.g., (Barbanoj et al., 2008; Duncan et al., 2013b). Moreover, most participants completed an adaptation night increasing the reliability of sleep findings, and the within-subject design allowed for a more efficient design in terms of statistical power. Second, our study aimed only at a short-term effect on sleep. It was thus not possible to detect any long-term sleep changes during the days following the administration or changes due to chronic or repeated psilocybin use. Third, the power spectral analysis of REM sleep could be influenced by the exclusion of phasic REM episodes which are characterized by eye movements. However, ensuring precise EEG spectral estimates free of confounding EOG artifacts was preferred.

In conclusion, our study is the first to document that psilocybin shows several expected sleep changes including increased REM sleep onset latency and a trend to decreased REM sleep duration following the first night after administration as documented in serotonergic antidepressants (especially SSRIs). Future research should explore psilocybin’s antidepressant properties using measures of mood or antidepressant response after administration and explore possible associations with sleep changes.

Our results did not confirm the assumption that psilocybin would increase delta power during SWS in the first sleep cycle as seen after ketamine administration (Duncan et al., 2017). Instead, psilocybin acted as other 5-HT_2_ receptor agonists that decrease SWS. Findings could reflect SWA alterations by mechanisms other than neuroplasticity. Overall, our study supports the potential role of psilocybin in sleep regulation and brings new insight into the clarification of its antidepressant properties.

## Data Availability Statement

The raw data supporting the conclusions of this article will be made available by the authors, without undue reservation, to any qualified researcher.

## Ethics Statement

The studies involving human participants were reviewed and approved by Ethical Committee of the National Institute of Mental Health. The patients/participants provided their written informed consent to participate in this study.

## Author Contributions

JH, TP, MB, FT, JKop, TF, DD, KJ, and MŠ contributed to the conception and design of the study; DD, KJ, MŠ, PZ, JKor, VA, MV, FT, and AB collected data; DD, KJ, MŠ, and ES organized and managed the dataset and performed the statistical analysis; JB, BM, and JKop contributed to the data analysis; DD, KJ, and MŠ wrote the first draft of the manuscript. All authors contributed to manuscript revision, read and approved the submitted version.

## Funding

This work was supported by the projects “Progres Q35”, 260533/SVV/2020, GACR grant no. 20-25349S, MICR grant no. VI20172020056, MHCR grant no. NV18-07-00272, MHCZ—DRO (NIMH-CZ, 00023752), project LO1611, with financial support from MEYS CR under the NPU I program.

## Conflict of Interest

The authors declare that the research was conducted in the absence of any commercial or financial relationships that could be construed as a potential conflict of interest.
